# Enhanced lactose utilization from milk enriched in Uridine-5’-monophosphate drives hepatic energy storage in young mammals

**DOI:** 10.1016/j.fochms.2026.100427

**Published:** 2026-06-01

**Authors:** Lu-min Gao, Lu Liu, Xu-dong Yang, Ci-min Long, Xin Wu

**Affiliations:** a Tianjin Institute of Industrial Biotechnology, Chinese Academy of Sciences, Tianjin 300308, PR China; b College of Advanced Agricultural Sciences, University of Chinese Academy of Science, Beijing 100049, China

**Keywords:** Uridine monophosphate, Nucleotides, Hepatic energy metabolism, Glycogen deposition, Lipogenesis

## Abstract

Lactose is the primary carbohydrate in milk, but its role in neonatal metabolic adaptation remains unclear. This study aimed to clarify the correlation between milk pyrimidine nucleotides and hepatic energy metabolism during lactose metabolism via three animal models. A sow-piglet model characterized natural milk nucleotide dynamics and their correlation with hepatic metabolism in suckling piglets. An ultra-early weaned piglet model evaluated uridine monophosphate (UMP) supplementation under controlled high-lactose conditions. A high-lactose rat model validated conserved metabolic alterations. Natural lactation analysis revealed that milk lactose increased obviously at days 7, 14 and 21 relative to colostrum, while milk pyrimidine nucleotides decreased gradually. Accordingly, suckling piglets exhibited increased serum triglyceride and decreased hepatic glycogen, together with suppressed expression of hepatic pyrimidine synthesis-related genes at later lactation stages. On this basis, we further explored the effects of UMP supplementation using high-lactose-fed piglets and rats. Dietary UMP supplementation was associated with increased hepatic glycogen and triglyceride accumulation in both species. UMP intervention coincided with increased activities of gluconeogenic and Leloir pathway enzymes, as well as altered expression of lipogenic genes. These changes were accompanied by changes in AMPK/mTOR, PPARγ and IL-6 signaling. Collectively, these results suggest that UMP supplementation was associated with hepatic energy storage in young mammals fed a lactose-containing diet.

## Highlights

• Milk pyrimidine nucleotide levels change in parallel with hepatic pyrimidine synthesis during neonatal lactation.
• UMP is associated with altered hepatic lactose-related energy storage in neonates.
• UMP correlates with variations in hepatic AMPK/mTOR, PPARγ, and IL-6 signaling profiles.

## Graphical abstract

**Figure:**
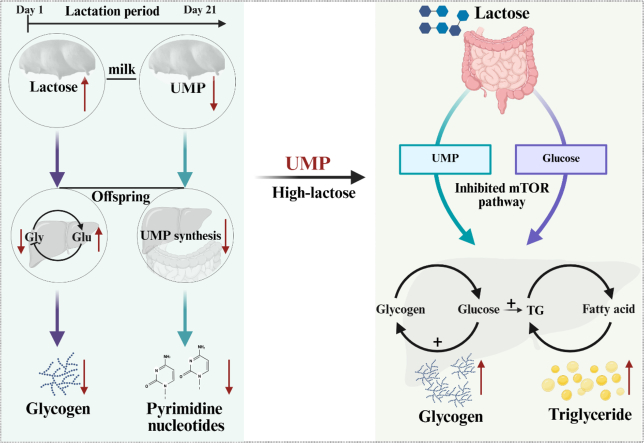

## 1 Introduction

As the primary carbohydrate in mammalian milk, lactose serves as a critical energy substrate and supports early-life metabolic homeostasis in neonates. It directly facilitates hepatic glycogen accumulation during the postnatal period (Brockway et al., 2024; Lara-Mota et al., 2021; Odell & Wallis, 2021), while the neonatal liver normally undergoes a metabolic shift shortly after birth, gradually shifting energy reserve preference from glycogen to lipids to sustain systemic glucose stability (Bakhronovna & Mukhlisakhan, 2025; Li et al., 2023). Although this adaptive metabolic shift is well recognized, the key factors associated with lactose utilization during this transition remain poorly understood. Elucidating the upstream factors linked to lactose metabolism is essential for understanding the physiological regulation of neonatal hepatic energy storage.

After intestinal hydrolysis, lactose is broken down into glucose and galactose, and galactose catabolism proceeds mainly through the Leloir pathway **(**Daenzer et al., 2024). This pathway requires uridine diphosphate-galactose (UDP-galactose), a derivative synthesized from uridine-5′-monophosphate (UMP), while UMP-derived UDP-glucose also acts as an essential substrate for hepatic glycogen synthesis (Yang & Li, 2024). These biochemical properties suggest a theoretical linkage between pyrimidine nucleotide level and lactose metabolism efficiency, whereas such theoretical association does not equal confirmed in vivo physiological function. Consistent with existing in vivo animal evidence, supplemental UMP has been shown to support the synthesis of UDP-galactose and UDP-glucose (DeVito et al., 2014; Hanssen et al., 2023), and may alleviate metabolic disorders induced by impaired UDP-sugar metabolism (Ng et al., 2015). Additionally, UMP is a predominant bioactive nucleotide in breast milk, and milk nucleotide profiles change dynamically during lactation (Ferreira, 2003; Hodgkinson et al., 2022; Mateo et al., 2004). Accumulating correlational studies have revealed that nucleotides are associated with hepatic glycolipid homeostasis in neonatal animals (Le et al., 2013; Nwosu et al., 2023; Tian et al., 2025). In particular, UMP shows apparent associations with glucose and lipid metabolic processes (Sahu et al., 2024; Zhang, et al., 2025), which hints at a possible relevance between UMP and neonatal lactose-related metabolic adaptation in neonates.

Despite these advances, few studies have examined whether milk pyrimidine nucleotides correlate with lactose utilization and hepatic energy metabolism in neonates. The relationship between milk lactose and nucleotide dynamics during lactation also remains unclear. We therefore hypothesized that UMP may be associated with lactose utilization and hepatic energy metabolism in young animals, and aimed to investigate this association and the potential role of milk nucleotides in early-life metabolic regulation.

## 2 Material and methods

### 2.1 Ethical approval

All animal experiments were approved by the Animal Care Committee of the Institute of Subtropical Agriculture, Chinese Academy of Sciences (approval nos. ISA-2021-0056, ISA-2022-0060, and ISA-2022-0059) and conducted in strict accordance with institutional animal welfare guidelines.

### 2.2 Experimental design and sample collection

**Fig. 1:**
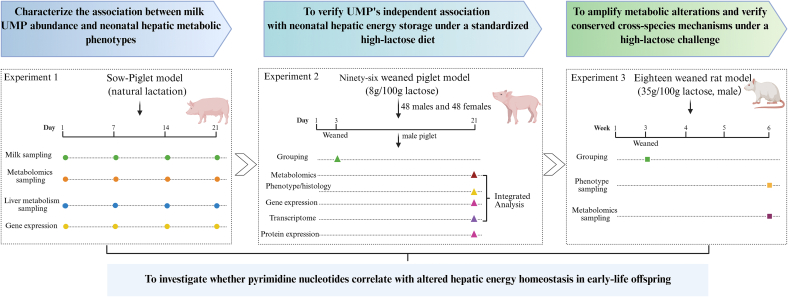
Schematic overview of the experimental design.

Three complementary animal models were used for cross-system validation and to strengthen conclusion reliability. Experiment 1 served as a sow-piglet observational model to characterize the physiological association between milk UMP abundance and neonatal hepatic metabolic phenotypes. Experiment 2 used ultra-early weaned piglets fed a standardized high-lactose diet to verify the independent metabolic association of UMP supplementation with neonatal hepatic energy metabolism, excluding maternal interference. Experiment 3 adopted a high-lactose rat challenge model to amplify metabolic alterations, verify conserved cross-species mechanisms, and facilitate pathway exploration (Fig. 1). Experiment 1 was an observational cohort study, while Experiments 2 and 3 were randomized controlled intervention trials with control and UMP-supplemented groups. Experimental units were clearly defined as litter for the observational study, pen as a nested factor for piglet trials, and individual animal for rat experiments.

#### 2.2.1 Experiment 1: Sow-piglet observational study

Seven pregnant sows (Large White × Landrace, parity 3–6) were housed in individual farrowing crates (2.2 m × 0.6 m) under controlled environmental conditions (22 ± 2 °C, 60 ± 5% humidity). Sows had ad libitum access to water and were fed a corn-soybean diet meeting NRC (2012) requirements (**Table S1**). Litter size was standardized after farrowing, and piglets remained with sows throughout lactation. Colostrum was collected within 12 h of farrowing, and milk on days 7, 14, and 21 of lactation, following oxytocin injection (10 IU, intramuscular). At each time point, one male piglet per litter was selected for sample collection. To minimize confounding effects of female hormonal cycles on metabolic parameters, only male piglets were used, and body weight was matched to the litter average to reduce inter-individual variation (Zhang, et al., 2025). Following a 12 h fast, piglets were euthanized by electrical stunning and exsanguination (AVMA guidelines). Blood was collected via cardiac puncture, centrifuged (3000 ×*g*, 15 min, room temperature), and serum was stored at −80 °C. Liver tissues were snap-frozen in liquid nitrogen and stored at −80 °C.

Only healthy piglets with normal birth weight and no congenital abnormalities were included. Exclusion criteria: birth weight < 1.3 kg or > 1.8 kg, or failure to suckle within 12 h post-birth. No animals were excluded during the study.

Sample size was calculated a priori using G*Power (version 3.1.9.7) based on effect sizes from previous studies (Mateo et al., 2004). Primary outcomes included milk lactose and UMP concentrations and hepatic glycogen content. Secondary outcomes included serum metabolites and hepatic gene expression.

#### 2.2.2 Experiment 2: Ultra-early weaned piglet experiment

Ninety-six healthy 3-day-old weaned piglets (Duroc × Landrace × Yorkshire, 48 males and 48 females; initial body weight 2.3 ± 0.2 kg) were housed in mixed-sex groups of 6 per pen (2.1 m × 1.8 m) on fully slatted plastic floors under controlled environmental conditions (25 ± 2 °C, 15 h light/9 h dark cycle) with localized heating lamps. Piglets had free access to water and were fed milk replacer every 1.5 h starting at 7:00 AM. The study was a controlled, parallel-group animal experiment with two dietary intervention arms: The control group (CON), and the treated group (UMP). Diarrhea parameters were recorded, whereas metabolic and molecular outcomes were assessed at the end of the intervention.

Only healthy piglets with weight 2.0–2.5 kg and no clinical signs of disease were included. Exclusion criteria: diarrhea (fecal score ≥ 3 on a 0–3 scale) or body weight < 1.8 kg or > 2.8 kg. No animals were excluded during the study.

Sample size was determined a priori using G*Power (version 3.1.9.7) based on expected effect sizes for hepatic glycogen changes (primary outcomes) based on preliminary data. A total of 8 animals per group provide 80% power at α = 0.05. Hepatic glycogen and triglyceride content were defined as primary outcomes; serum metabolites, hepatic gene expression, and histomorphology were secondary outcomes. Omics analyses were defined as exploratory outcomes.

Upon arrival at the animal facility, piglets were stratified by initial body weight and sex, then randomly assigned to pens by an independent researcher not involved in the experiment. Pens were randomly distributed within the barn, and all piglets within the same pen received the same dietary intervention. Due to the nature of oral gavage administration, blinding during treatment administration was not feasible. However, investigators performing biochemical, molecular, histological, and statistical analyses were blinded to group allocation as samples were identified by numeric codes. The UMP and control solutions were identical in appearance.

Both groups received a basal milk replacer formulated to meet NRC (2012) requirements, with nutritional composition of 8.0% lactose, 2.7% fat, and 4.1% protein (**Table S2**). The CON group (*n* = 8, 6 piglets/pen) received the basal milk replacer, while the UMP group (n = 8, 6 piglets/pen) received the same diet supplemented with 650 mg UMP—Na₂ per piglet per day (∼178 mg/kg body weight/day). UMP—Na₂ (purity ≥99.80%, molecular weight: 368.14 g/mol; Meiya Haian Pharmaceutical, Hangzhou, China) was dissolved in water and administered by oral gavage twice daily. This dose was selected based on the observed decline in milk UMP concentration during lactation and average daily milk intake, as well as previous literature (Li et al., 2019).

After the 18-day intervention, one male piglet per pen was selected for sample collection. Body weight was matched to the pen average to ensure consistent biological replicates, resulting in eight replicates for the CON group and eight for the UMP group (*n* = 8 per group). Only male piglets were used to avoid confounding effects of female hormonal cycles on hepatic energy metabolism. After a 12 h fasting period, blood samples (5 mL) of selected piglets were collected aseptically via cardiac puncture into vacuum tubes without heparin, and centrifuged at 3000 ×*g* for 15 min at room temperature to obtain serum, which was stored at −80 °C. Following blood collection, piglets were euthanized by electrical stunning followed by exsanguination, in accordance with institutional animal welfare guidelines. Liver tissues were then immediately collected. A portion was flash-frozen in liquid nitrogen and stored at −80 °C for molecular analyses, while another portion was fixed in 10% formalin (pH 7.3 ± 0.2) and glutaraldehyde for histological and electron microscopy.

#### 2.2.3 Experiment 3: Rat experiments

Eighteen three-week-old male Sprague-Dawley rats were purchased from SLAC Laboratory Animal Central (Changsha, China). Rats were individually housed in an air-conditioned specific-pathogen-free room (22 ± 2 °C, 50 ± 5% humidity, 12 h light/12 h dark cycle) with wood-chip bedding. Prior to the intervention, rats had ad libitum access to standard AIN-93G diet and filtered tap water. The study was a controlled, parallel-group animal experiment with two dietary intervention arms: (1) high-lactose diet (HLD) group – AIN-93G diet containing 35% lactose; (2) UMP group – HLD supplemented with 600 mg/kg UMP—Na₂. The individual rat was considered the experimental unit as the primary outcomes were not affected by cage effects (all *P* > 0.05 in two-way ANOVA tests). Body weight and intake parameters were recorded longitudinally, whereas metabolic, and molecular outcomes were assessed cross-sectionally at the end of the intervention.

Only healthy, normoglycemic male rats (fasting glucose <110 mg/dL) with initial body weight 120–140 g were included. Exclusion criteria: signs of illness or diarrhea before treatment. No animals were excluded during the study.

Sample size was determined a priori using G*Power based on expected effect sizes for hepatic triglyceride and glycogen (primary outcomes) based in previous studies. A total of 18 rats provide ≥80% power to detect relevant differences at α = 0.05. Hepatic glycogen and triglyceride content were defined as primary outcomes; serum metabolites, and hepatic gene expression were secondary outcomes. Omics analyses (transcriptomics, metabolomics) were defined as exploratory outcomes.

Upon arrival, rats were acclimated for one week. Prior to the intervention, fasting blood glucose was measured to confirm normoglycemia, and animals were randomly assigned to cages by facility personnel not involved in the experiment. Cage-mates received the same diet. While the dietary intervention precluded blinding during treatment, all subsequent analyses (biochemical, molecular, histological, and statistical) were performed blinded to group allocation using numeric codes for animal identification.

Rats in the HLD group were fed an AIN-93G diet containing 35% lactose, while the UMP group received the same diet supplemented with 600 mg/kg UMP—Na₂ (equimolar to 400 mg/kg uridine; molecular weight: 244.20 g/mol) (Liu et al., 2019; Liu et al., 2021). The lactose challenge protocol was adapted from a previously described method (Teichenné et al., 2023). Detailed diet composition is provided in **Table S3**. After three weeks of ad libitum feeding and an overnight fast, rats were euthanized under isoflurane anesthesia. Blood (10 mL) was collected from the abdominal aorta using a heparin-coated syringe, centrifuged (3000 ×*g*, 15 min, room temperature), and serum stored at −80 °C. Liver tissues were immediately collected and processed as follows: a portion was fixed in 4% neutral buffered formalin or 2.5% glutaraldehyde and stored at 4 °C for histological and electron microscopic examination; another portion was collected and preserved in liquid nitrogen at −80 °C until further analysis.

### 2.3 Sample analysis

All analyses were performed under a unified hierarchical framework, in which phenotypic and biochemical data served as primary evidence, histological and molecular profiles as supporting mechanistic evidence, and multi-omics results as exploratory evidence. Individual animal samples represented biological replicates, while all biochemical and molecular assays were conducted with technical replicates to ensure reproducibility.

#### 2.3.1 Milk nutrients analysis

Milk samples from 7 sows (*n* = 7) were collected for nutrient analysis. Lactose content was measured using a MilkoScan FT120 infrared automatic analyzer (Foss, Hillerød, Denmark). Nucleotides were quantified by HPLC (Agilent 1200, Agilent Technologies, Santa Clara, CA, USA) in accordance with national standard protocols, with quantification based on standard calibration curves.

#### 2.3.2 Untargeted metabolomic analysis

Serum biological replicates from suckling piglets (n = 7) and weaned piglets (*n* = 8 per group) were used for untargeted metabolomics. Serum was extracted with ice-cold methanol:acetonitrile (1:1, *v*/v) with internal standards, sonicated, and centrifuged. The supernatant was filtered and analyzed by LC-MS/MS using an Orbitrap QExactive HF mass spectrometer and Vanquish UHPLC system (Thermo Fisher), provided by Biotree (Shanghai, China). Pooled QC samples were injected throughout the analytical run. Unsupervised principal component analysis (PCA) was first performed to assess overall metabolic variation. Orthogonal partial least-squares discriminant analysis (OPLS-DA) was used to identify features contributing to group separation. Seven-fold cross-validation and 200-time permutation tests were performed to assess model stability. Differential metabolites were identified using a unified threshold (|log₂FC| > 1, FDR-adjusted *P* < 0.05, VIP ≥ 1.0). Functional annotation and pathway enrichment were performed using the Kyoto Encyclopedia of Genes and Genomes (KEGG) database.

#### 2.3.3 UHPLC-MS analysis and metabolite identification

Hepatic samples from 6 rats per group (*n* = 6) were analyzed using UHPLC-MS. Briefly, samples were sonicated in ice bath for 60 min with 1 mL extraction solution (acetonitrile:methanol:water = 2:2:1, *v*/v/v). After 1 h incubation at 20 °C, the supernatant was collected by centrifugation (12,000 rpm, 13,800 ×g, 15 min, 4 °C) for LC-MS analysis. UHPLC separation was performed on an Agilent 1290 Infinity system equipped with a BEH Amide column (2.1 × 100 mm, 1.7 μm). Mobile phase A was 25 mmol/L ammonium acetate and 25 mmol/L ammonium hydroxide in water (pH 9.75), and mobile phase B was acetonitrile. The gradient was: 0–1.0 min (95% B), 1.0–14.0 min (95–65% B), 14.0–16.0 min (65–40% B), 16.0–18.0 min (40% B), 18.0–18.1 min (40–95% B), 18.1–23.0 min (95% B). Mass spectrometry was performed in positive and negative electrospray ionization (ESI) modes, with QC samples included throughout the run.

MRM-based quantification was performed according to established methods (Cao et al., 2020) using the MT2000 Kit (Biotree, Shanghai, China). Peak areas were integrated with MultiQuant 3.0.2. PCA was first performed to visualize overall metabolic variation. PLS—DA and OPLS-DA were used as exploratory tools to identify metabolites contributing to group separation. Permutation tests (*n* = 200) were performed, and the Q^2^ intercept was evaluated for potential overfitting. Multiple testing was corrected using Benjamini-Hochberg FDR, and metabolic pathway analysis was performed using KEGG database.

#### 2.3.4 TC and TG analysis

Serum glucose, total cholesterol (TC) and triglyceride (TG), high density lipoprotein (HDL) and low-density lipoprotein (LDL) were measured using an automated biochemistry analyzer (Synchron CX Pro, Beckman Coulter, Fullerton, CA, USA) with kits from Beijing Chemlin Biotech Co., Ltd. (Beijing, China). Hepatic glucose, glycogen, TC and TG were measured using commercial kits (Nanjing Jiancheng Bioengineering Institute, Nanjing, China). Biological replicates: suckling piglets (*n* = 7), weaned piglets (*n* = 8), rats (*n* = 9 per group). All biochemical measurements were detected in duplicate technical replicates, and the average value was used for subsequent analysis.

#### 2.3.5 Histological analysis

Liver samples from 8 weaned piglets per group (n = 8) were fixed in 4% paraformaldehyde, embedded and stained with H&E. For each animal, three non-consecutive sections and five random microscopic fields were observed. All image analysis was completed by blinded researchers to avoid assessment bias.

#### 2.3.6 Transmission electron microscopy (TEM)

Hepatic samples of weaned piglets were subjected to TEM ultrastructural observation with three biological replicates per group (*n* = 3), which is a standard sample size for high-resolution tissue ultrastructural analysis in animal nutrition studies. Ultrastructural sample preparation and staining were performed following standard protocols, and examined using an HT7700 TEM (Hitachi, Tokyo, Japan). Three tissue blocks per animal were prepared, and five randomly selected fields per section were examined. All images were captured and analyzed by an investigator blinded to treatment groups.

#### 2.3.7 Medium- and long-chain FAs (MLCFAs) analysis

Serum and hepatic fatty acid profiles were determined in weaned piglets (*n* = 8) and rats (*n* = 9 per group). Samples were extracted overnight with 5% acetyl chloride in methanol at 50 °C, then mixed with n-hexane and centrifuged. Fatty acid methyl esters (FAMEs) in liver were prepared by lipid extraction with benzene-petroleum ether (1:1, *v*/v) for 24 h, followed by rapid methylation with 0.4 mol/L KOH in methanol. FAMEs were analyzed by gas chromatography (Agilent 6890, Boston, MA, USA), with a QC sample injected every 10 runs. All samples were analyzed in duplicate technical replicates.

#### 2.3.8 Hepatic glycogen staining (PAS)

Liver tissues from weaned piglets (*n* = 3 per group) were processed for PAS staining to visualize hepatic glycogen deposition. Histological staining and morphological observation were performed using standardized pathological procedures. Glycogen appeared as magenta-red granules in hepatocyte cytoplasm, with blue nuclei and a clear or light pink background.

#### 2.3.9 qRT- PCR analysis

Total RNA was extracted from liver tissues of suckling piglets (*n* = 7), weaned piglets (*n* = 8) and rats (*n* = 9 per group). Reverse transcription was performed using the PrimerScript RT kit (Takara, Shiga-ken, Japan) with 1 μg total RNA according to the manufacturer's protocol. Thermal cycling on a Bio-Rad iCycler (Bio-Rad, Hercules, CA, USA) was performed as follows: 95 °C for 10 min, then 40 cycles of 95 °C for 15 s and 60 °C for 50 s. Primers were designed with Primer 5 (Applied Biosystems, Foster City, CA, USA), and each pair yielded a single melting curve peak **(Table S4**). Relative gene expression was calculated using the 2^-ΔΔCt^ method with technical triplicates for each sample. β-Actin was used as the reference gene for normalization, and all experiments were repeated using a second endogenous control gene glyceraldehyde-3-phosphate dehydrogenase (GAPDH) to validate the results (data shown refer to normalization with β-actin only).

#### 2.3.10 Transcriptomic analysis

Transcriptome sequencing was conducted on hepatic tissues of weaned piglets using four biological replicates per group (*n* = 4), which is a commonly adopted sample size for exploratory transcriptomic research in piglet nutritional intervention trials. Briefly, total RNA was extracted using the RNAprep Pure Kit DP432 (TIANGEN Biotech, Beijing, China) according to the manufacturer's protocol. RNA integrity was assessed using a Qubit 4.0 fluorometer (Thermo Fisher Scientific, MA, USA) and 1.5% agarose gel electrophoresis. Sequencing libraries were prepared from 3 μg total RNA using the MGIEasy mRNA Library Prep Kit and sequenced as 100 bp paired-end reads on the DNBSEQ-T7 platform. Adapter sequences and low-quality reads were removed using Cutadapt v1.11, and clean reads were aligned to the pig reference genome (*Sus scrofa*; GCF_000003025.6_Sscrofa11.1) using Hisat2 v2.1.0. Gene annotation was performed against the RefSeq NR database, and transcript quantification was achieved using FeatureCounts v1.6.0, with expression levels normalized as FPKM. Differential expression analysis using EdgeR identified significant genes with |log₂FC| > 1 and FDR < 0.05. Functional enrichment of differentially expressed genes (DEGs) was analyzed via KEGG pathways, with significance determined by hypergeometric distribution (Q-value threshold = 0.05).

#### 2.3.11 Protein extraction and immunoblotting analysis

Hepatic biological replicates (*n* = 4 per group) were used for western blot detection. Total protein was extracted using RIPA lysis (Beyotime, P00113B, Shanghai, China) buffer containing protease and phosphatase inhibitors. Proteins were separated by SDS-PAGE, transferred onto PVDF membranes (Millipore, ISEQ00010, USA), and blocked with 5% BSA for 2 h at room temperature. Membranes were then incubated with primary and secondary antibodies (**Table S5**). Protein bands were visualized using Supersignal West Dura chemiluminescent substrate (Thermo Fisher Scientific, Waltham, MA, USA) and imaged on a chemiluminescence system (Applygen Technologies, Beijing, China). For automated capillary-based Western (WES), protein lysates were mixed with 5× fluorescent master mix and denatured by boiling for 5 min. Samples, along with wash buffer, primary antibodies, secondary antibodies, blocking reagent, and chemiluminescent substrate, were dispensed into a pre-defined microplate. Protein separation and immunodetection were automatically carried out in individual capillaries using default instrument settings. Band intensities were quantified using ImageJ software (NIH, Bethesda, MD, USA). WES data were analyzed using Compass software 3.1 (ProteinSimple, San Jose, CA, USA). Relative protein expression was calculated as fold change vs. control. Experiments were repeated three times independently.

### 2.4 Statistics

All statistical analyses were conducted using SPSS 22.0, GraphPad Prism 6.0, and R software (v4.2.0). Normality and homogeneity of variances were assessed using the Shapiro-Wilk test and Levene's test, respectively. Data are presented as mean ± SEM, with statistical significance set at *P* < 0.05.

For longitudinal milk profiling in the sow-piglet observational model, repeated-measures ANOVA with Bonferroni post hoc correction was applied, with sampling time as a fixed effect and individual sow/litter as the experimental unit. For multi-time-point biochemical and gene expression profiles in suckling piglets, one-way ANOVA with Tukey's b post hoc test was performed, with litter as the random nesting factor to match the defined experimental unit.

For two-group comparisons in ultra-early weaned piglet and rat intervention experiments, unpaired Student's *t*-test or Mann-Whitney test was selected according to data normality. Pen effect was included as a random effect for piglet data, while individual animal was defined as the independent experimental unit for rat analyses. Cohen's d effect sizes were calculated for all key phenotypic comparisons to quantify the magnitude of treatment differences.

Spearman correlation analyses were performed using R (v4.2.0) to identify associations between differential metabolites and differential genes. To reduce false positives from multiple testing, the Benjamini-Hochberg FDR correction was applied, and only correlations with adjusted *P* < 0.05 were considered. To facilitate integrated analysis beyond independent correlation screening, we further filtered significant gene-metabolite pairs and retained those jointly annotated to glucose and lipid metabolism pathways according to KEGG databases, followed by the construction of multi-omics association networks.

## 3 Results

### 3.1 Lactation-associated metabolic characteristics in suckling piglets

#### 3.1.1 Dynamic changes in milk components during the natural lactation

**Fig. 2:**
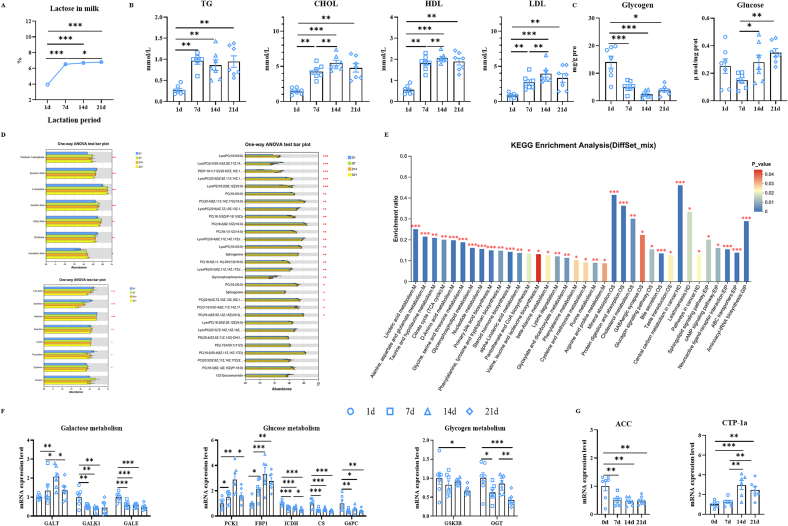
**Hepatic metabolic profiles and dynamic milk nutrient changes during natural lactation in suckling piglets.** (A) Lactose in milk of sow. (B) Serum lipid profiles in suckling piglet. (C) Hepatic glucose and glycogen levels in suckling piglet. (D) Serum metabolite levels related to glucose, lipid, and nucleotide metabolism in suckling piglet. (E) KEGG pathway enrichment analysis of serum differential metabolites. (F) Hepatic expression of key enzymes in glucose and galactose metabolism. (G) Hepatic expression of key enzymes in triglyceride metabolism. Data are shown as mean ± SEM (*n* = 7). *P < 0.05, **P < 0.01, ***P < 0.001.

As lactation progressed and piglets matured, lactose concentration significantly increased at days 7, 14 and 21 relative to day 1 colostrum (*P* < 0.01; Fig. 2A). Conversely, the abundance of pyrimidine nucleotides in milk decreased gradually throughout lactation (*P* < 0.05; Table 1).

**Table 1:** Nucleotide profile in milk (μg/mL).

Item	Days of lactation				*P-*value
	1d	7d	14d	21d	
UMP-Na₂ + CMP-Na₂	445 ± 43 ^A^	267 ± 23^B^	230 ± 35^BC^	175 ± 28^C^	< 0.001
AMP-Na₂ + GMP-Na₂ + IMP-Na₂	69 ± 13^a^	109 ± 8 ^b^	95 ± 16^ab^	160 ± 10^c^	0.001
Total Nucleotides	758 ± 79 ^A^	564 ± 36 ^B^	474 ± 70 ^B^	447 ± 38 ^B^	0.006

Data are presented as mean ± SEM (*n* = 7). Data are presented as mean ± SEM (n = 7). Different superscript letters indicate significant differences (a, b, c: *P* < 0.05; A, B, C: *P* < 0.01).

#### 3.1.2 Metabolic characteristics of serum

Alongside lactation progression and piglet physiological development, temporal alterations were observed in the serum metabolic profile (**Fig. S1**). Differentially metabolites were mainly enriched in nucleotide, carbohydrate and lipid metabolism pathways (Fig. 2D-E).

#### 3.1.3 Hepatic glucose metabolism

Hepatic glycogen levels were lower on days 7, 14, and 21 compared to day 1 (*P* < 0.05; Fig. 2E), whereas hepatic glucose level was higher on days 14 and 21 compared to day 7 (*P* < 0.05).

The mRNA levels of glucose, galactose, and glycogen metabolism-related (*GALK1*, *GALE*, *ICDH*, *CS*, and *G6PC* declined throughout lactation, with lower O linked N acetylglucosamine transferase (OGT*)* and glycogen synthase kinase 3 beta (GSK3B) expression observed on day 21 relative to day 1 (*P* < 0.05). The transcript levels of galactose-1-phosphate uridylyltransferase (GALT), phosphoenolpyruvate carboxykinase 1 (PCK1), and fructose-1,6-bisphosphatase 1 (FBP1) peaked on day 14 and were significantly higher than those at other lactation stages (*P* < 0.05; Fig. 2F).

#### 3.1.4 Hepatic lipid metabolism

Serum TG, TC, HDL, and LDL concentrations were higher at days 7, 14, and 21 of lactation than at the early lactation stage (*P* < 0.01; Fig. 2B). Hepatic acetyl-CoA carboxylase (ACC) expression was downregulated, while carnitine palmitoyltransferase 1a (CPT1a) expression was upregulated at day 7, 14 and 21 (*P* < 0.05; Fig. 2G).

#### 3.1.5 Hepatic pyrimidine synthesis-related genes expression

Milk and serum pyrimidine nucleotide levels declined across lactation, with synchronous temporal changes in the expression of hepatic pyrimidine synthesis-related genes. The hepatic expression of key pyrimidine synthesis enzymes (*CAD*, *UMPS*, *CDA*, *UCK1*, *CTPS1*, and *CTPS2*) was decreased during natural lactation (*P* < 0.05; Table 2).

**Table 2:** Hepatic mRNA expression of key enzymes involved in de novo pyrimidine synthesis in suckling piglets.

Item	Days of lactation				*P-*value
	1d	7d	14d	21d	
UMPS	1.00 ± 0.14 ^A^	0.48 ± 0.06^B^	0.52 ± 0.04^B^	0.50 ± 0.09^B^	0.001
CAD	1.00 ± 0.11 ^A^	0.45 ± 0.08^B^	0.40 ± 0.03^B^	0.25 ± 0.02^B^	<0.001
CDA	1.00 ± 0.240 ^A^	0.35 ± 0.04^B^	0.48 ± 0.06^B^	0.62 ± 0.04^B^	0.008
UPP2	1.00 ± 0.27	1.54 ± 0.59	1.04 ± 0.15	1.75 ± 0.25	0.37
UCK1	1.00 ± 0.12^a^	0.80 ± 0.08^ac^	0.72 ± 0.05^c^	0.52 ± 0.11^b^	0.012
CTPS1	1.00 ± 1.11 ^A^	0.51 ± 0.11^B^	0.44 ± 0.08^B^	0.29 ± 0.05 ^B^	0.000
CTPS2	1.00 ± 0.21^Aa^	0.57 ± 0.05^b^	0.62 ± 0.04^b^	0.25 ± 0.05^Bb^	0.001
UCP2	1.00 ± 0.28^a^	1.14 ± 0.32^a^	0.57 ± 0.12^b^	0.13 ± 0.01^b^	0.015
CMPK1	1.00 ± 0.13^a^	1.19 ± 0.19^a^	1.78 ± 0.21^b^	1.44 ± 0.25^ab^	0.06
CMPK2	1.00 ± 0.17 ^A^	8.01 ± 1.36^B^	10.12 ± 2.44^B^	10.74 ± 2.77^B^	0.007

Data are presented as mean ± SEM (n = 7). Data are presented as mean ± SEM (n = 7). Different superscript letters indicate significant differences (a, b, c: *P* < 0.05; A, B: *P* < 0.001).

### 3.2 Effect of UMP on hepatic glycogen deposition in weaned piglets and rats

Hepatic glycogen-related phenotypic alterations were compared between UMP-supplemented and control weaned piglets and high-lactose-fed rats.

#### 3.2.1 Serum carbohydrate metabolites

**Fig. 3:**
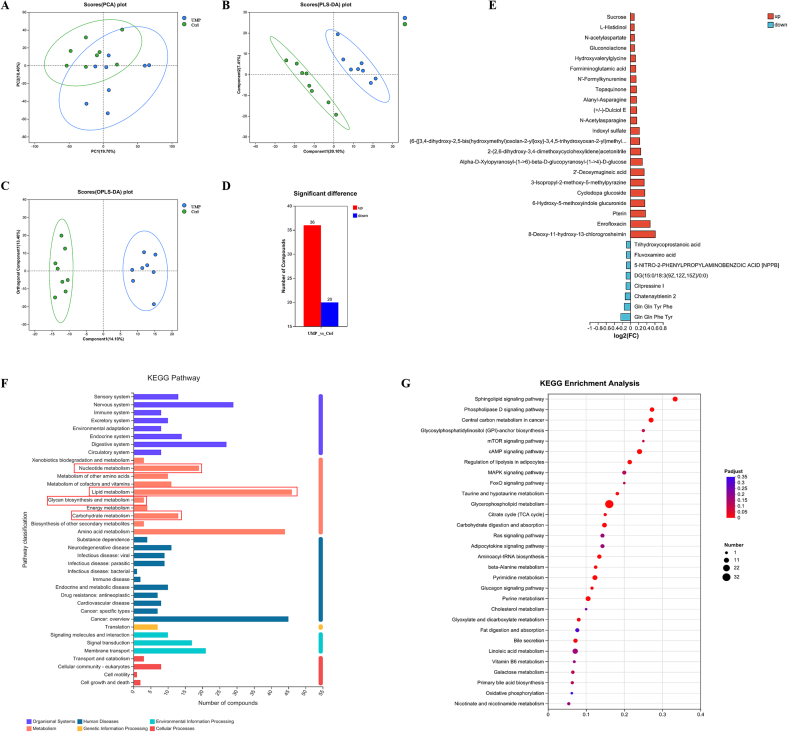
**Serum metabolite profiles associated with UMP supplementation in weaned piglets.** (A-D) Multivariate analysis of serum metabolites (PCA, PLS-DA, OPLS-DA). (E) Differential serum metabolites. (F, G) KEGG pathway enrichment analysis of differential metabolites.

PCA and multivariate analysis revealed distinct serum carbohydrate metabolic profiles in UMP-supplemented piglets relative to controls (Fig. 3A-D**; Fig. S2**). At the metabolite level in the serum, UMP supplementation was accompanied by higher serum glucose, succinic acid, and gluconolactone levels and lower fructose 1-phosphate levels (Fig. 3E). Differential metabolites were enriched in carbohydrate digestion, TCA cycle, and pyrimidine metabolism pathways (Fig. 3F-G).

#### 3.2.2 Hepatic glycogen deposition

**Fig. 4:**
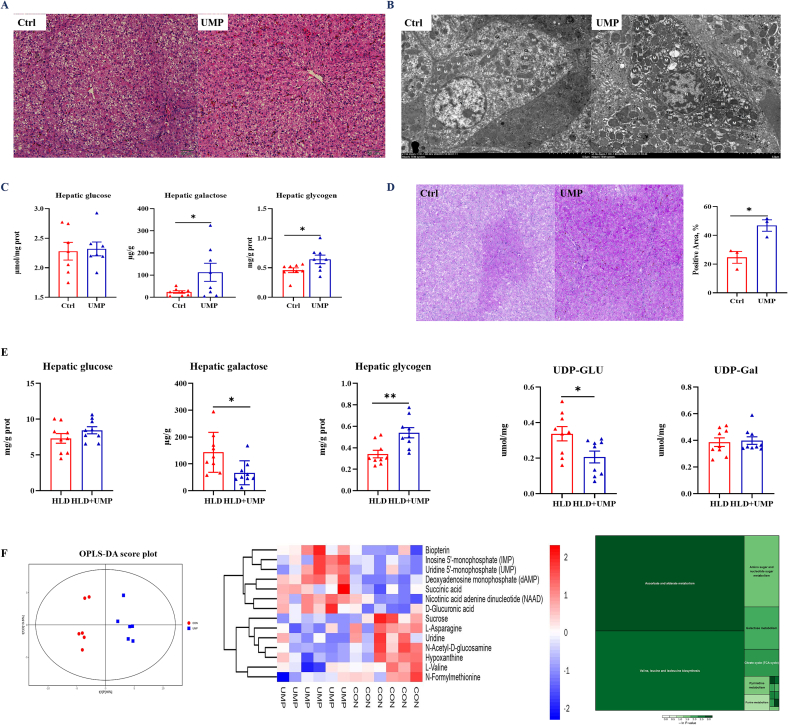
Associations between UMP supplementation and hepatic glycogen deposition in weaned piglets and rats fed a high-lactose diet. (A, B) Representative images of hepatic H&E staining and TEM in weaned piglets. (C) Hepatic glucose, galactose, and glycogen levels in piglets. (D) Representative PAS staining of hepatic tissue in weaned piglets. (E) Hepatic glucose, galactose, glycogen, UDP-glucose, and UDP-galactose in rats. (F) Targeted metabolites related to central carbon metabolism in the liver of rats. Data are presented as mean ± SEM (*n* = 3–8). Statistical significance is denoted as *P < 0.05, and **P < 0.01.

No differences in hepatic ultrastructure (mitochondria and rough endoplasmic reticulum) or serum alanine aminotransferase (ALT) levels were observed between groups (*P* > 0.05, **Fig. S3**). At the phenotypic level, UMP-treated piglets exhibited decreased serum aspartate aminotransferase (AST) levels, and increased hepatic glycogen deposition, which was further validated by PAS staining, accompanied by increased hepatic galactose accumulation (*P* < 0.05; Fig. 4**A-D**; Fig. **S3**). In rats fed a 35% high-lactose diet, UMP supplementation increased hepatic glycogen levels and reduced hepatic galactose and UDP glucose levels (*P* < 0.05; Fig. 4E).

#### 3.2.3 Hepatic central carbon metabolism

To further characterize hepatic metabolic alterations at the molecular level, targeted metabolomics identified 14 differentially abundant hepatic metabolites following UMP treatment, including nucleotide salvage intermediates (IMP, UMP, dAMP, UR) and TCA cycle-related metabolites. These metabolites were mainly enriched in the TCA cycle, galactose metabolism, and nucleotide sugar metabolism pathways (Fig. 4**F, S5**).

### 3.3 Effect of UMP on hepatic TG deposition and fatty acid composition

**Fig. 5:**
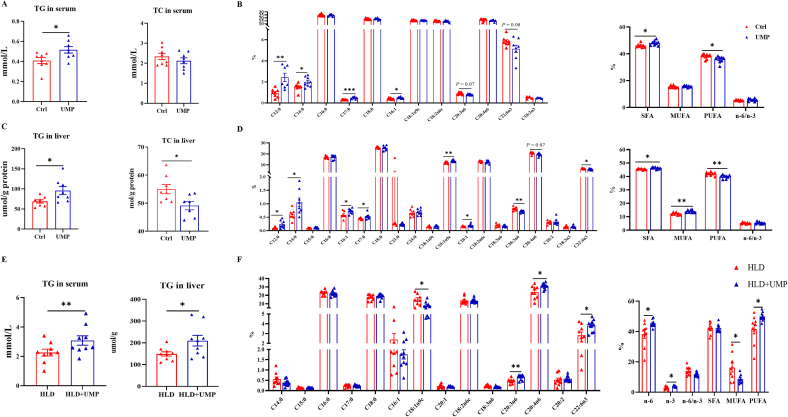
**Species-specific hepatic lipid profiles associated with UMP supplementation in weaned piglets and rats fed a high-lactose diet.** (A) Serum TC and TG in piglets. (B) Serum fatty acid composition in piglets. (C) Hepatic TC and TG in piglets. (D) Hepatic fatty acid composition in piglets. (E) Serum and hepatic TG level in rat. (F) Hepatic fatty acid composition in rat. Data are presented as mean ± SEM, *n* = 8–9. Statistical significances were set at *P < 0.05, **P < 0.01.

UMP was associated with higher serum and hepatic TG levels in both piglets and rats, and lower hepatic TC in piglets (*P* < 0.05; Fig. 5A, C, E). In piglet serum, UMP supplementation increased the levels of medium-chain saturated fatty acids (C12:0, C14:0, C16:1, C17:0) and decreased C20:3n6 levels. The liver of UMP-treated piglets showed higher saturated fatty acids (SFAs) and monounsaturated fatty acids (MUFAs) levels and lower polyunsaturated fatty acids (PUFAs) levels (*P* < 0.05; Fig. 5B-D). In rats, UMP was associated with higher hepatic long-chain PUFAs (C20:4n6, C20:3n6, C22:6n3) and lower MUFA C18:1n9c (*P* < 0.05; Fig. 5F).

### 3.4 Correlation-based analysis of UMP-associated glycogen and TG deposition

#### 3.4.1 Hepatic glucose and galactose metabolism

**Fig. 6:**
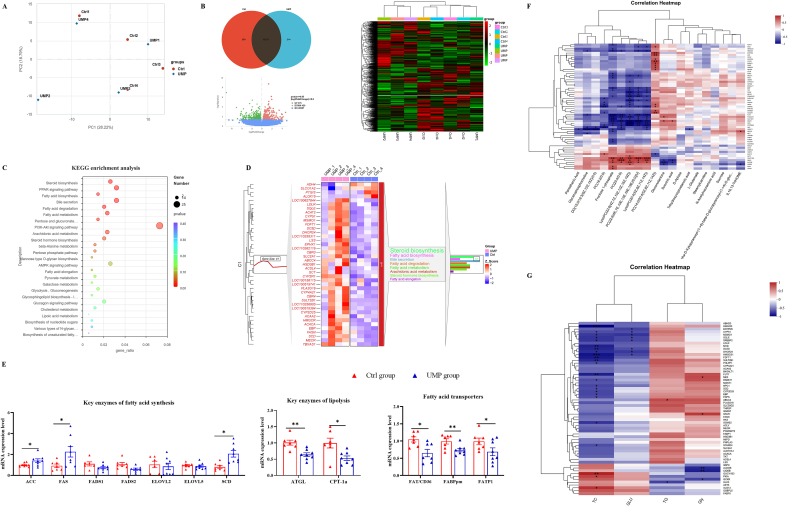
**Hepatic transcriptomic changes associated with UMP supplementation in weaned piglets.** (A) PCA score plot. (B) Numbers of DEGs associated with UMP. (C) KEGG pathway enrichment of DEGs; (D) KEGG pathway enrichment of lipid-metabolism-related DEGs. (E) Expression of key lipid-metabolism-related genes. (F) Correlation between differential serum metabolites and differential hepatic genes. (G) Correlation between hepatic glucose, triglyceride, cholesterol, glycogen levels and differential hepatic genes. Data are presented as mean ± SEM (*n* = 4–8). Statistical significance is denoted as *P < 0.05, and **P < 0.01.

**Fig. 7:**
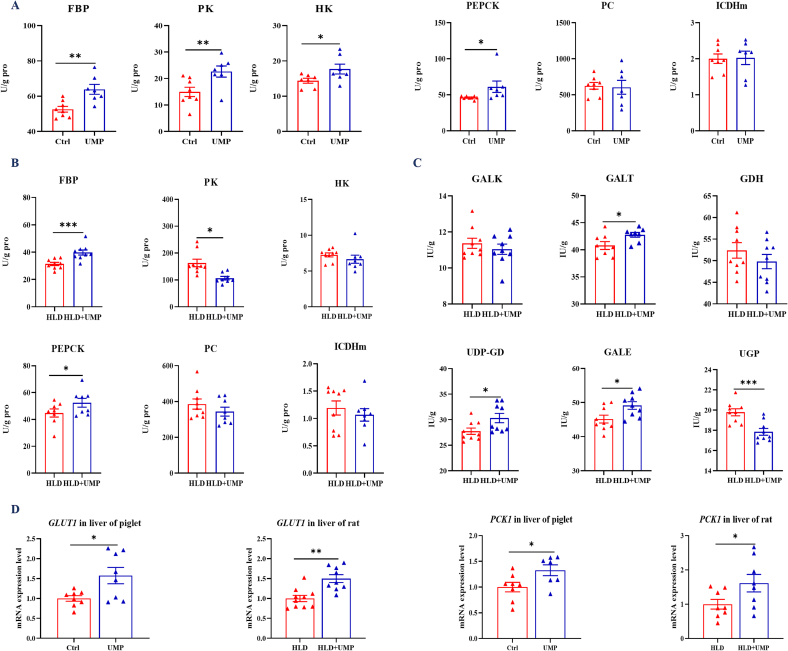
Associations between UMP supplementation and hepatic glucose and galactose metabolism in weaned piglets and rats fed a high-lactose diet. (A) key enzyme activities in glucose metabolism in piglet. (B) key enzyme activities in glucose metabolism in rats. (C) Key enzyme activities in galactose metabolism in rat. (D) Gene expression of *GLUT1* and *PCK1* in piglets. Statistical significance is denoted as *P < 0.05, **P < 0.01, and ***P < 0.001.

Differential gene expression and enzyme activity changes related to glucose and galactose metabolism were observed after UMP intervention. At the transcriptomic level, genes involved in glucose uptake and galactose metabolism (*SLC2A1*, *B4GALT1*, *UGDH*, *HK3*) were upregulated. (Fig. 6A-D). At the enzymatic activity level, UMP supplementation increased the activities of gluconeogenic enzymes (fructose-1,6-bisphosphatase (F-1,6-BP) and phosphoenolpyruvate carboxykinase (PEPCK) (*P* < 0.05; Fig. 7A, D). By contrast, species-specific glycolytic changes were observed, with higher hexokinase (HK) and pyruvate kinase (PK) activities in piglets and lower pyruvate kinase activity in rats (*P* < 0.05; Fig. 7A, D). UMP also associated with higher Leloir pathway enzyme activities (GALT, GALE, UDP-GD) and lower UDP-glucose pyrophosphorylase (UGP) activity (*P* < 0.05, Fig. 7F). Independent qPCR confirmed higher expression of *GLUT1* and *PCK1* (*P* < 0.05, Fig. 7D).

#### 3.4.2 Hepatic lipid metabolism

Transcriptomic changes were enriched in lipid metabolism pathways, the genes associated with lipid anabolism (*ACACA*, *FASN*, *SCD*, *MECR*) were upregulated following UMP supplementation (Fig. 6C-D). By contrast, the expression of lipid transport genes (*FAT/CD36*, *FABPpm*, *FATP1*) and lipolytic genes (*ATGL*, *CPT1a*) was downregulated (*P* < 0.05; Fig. 7B).

#### 3.4.3 Correlation analyses

Pearson correlation analysis revealed significant statistical associations between metabolic phenotypes and gene expression profiles (Fig. 6F-G). Hepatic TC was negatively correlated with cholesterol synthesis-related genes (*SC5D*, *HMGCS1*, *MVD*, *FDFT1*, *SULT2B1*, *CYP2D25*, *SCD*), while TG level was positively correlated with *ABCC4* and *PCK1* (*P* < 0.05; Fig. 6G). Serum fructose 1-phosphate was positively correlated with glucose metabolism gene (*PKIA*, *GCKR*, *ALDOB*, *G6PC*, *GUCY1B3*), whereas succinic acid was negatively correlated with *ALDOB* and *G6PC* (*P* < 0.05; Fig. 6F).

### 3.5 Effect of UMP on hepatic signaling pathways

**Fig. 8:**
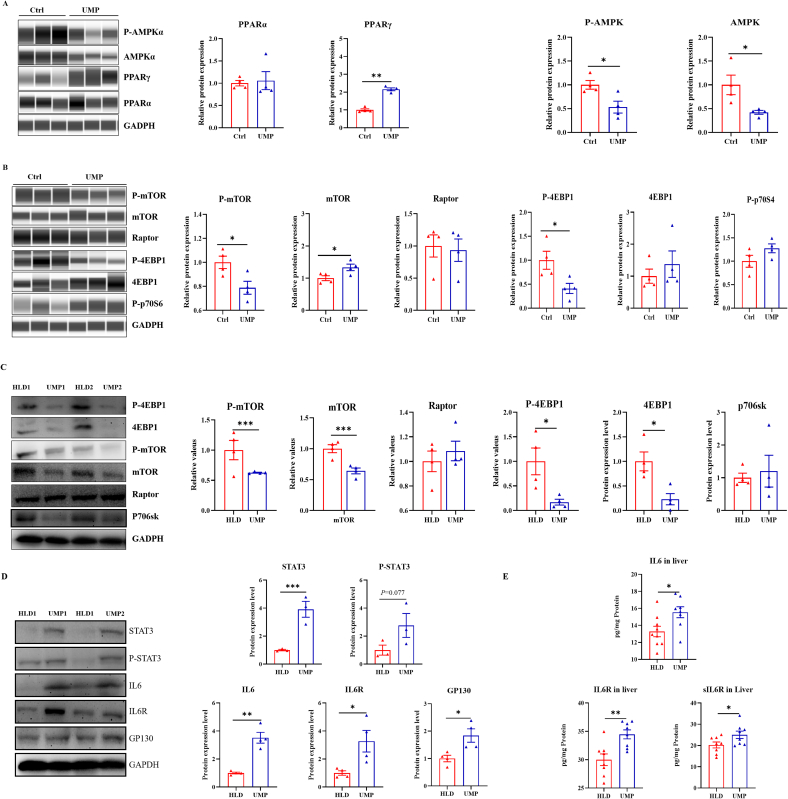
Hepatic signaling pathway alterations associated with UMP supplementation in weaned piglets and rats. (A) PPARs and AMPK protein expression in piglets. (B, C) mTOR pathway protein expression in piglets (B) and rats (C). (D, E) IL-6/STAT3 pathway protein expression in rats. Data are presented as mean ± SEM (n = 3–9). Statistical significance is denoted as *P < 0.05, **P < 0.01, and ***P < 0.001.

Transcriptomic changes were enriched in the phosphatidylinositol 3-kinase (PI3K)-Akt, peroxisome proliferator-activated receptors (PPARs), mechanistic target of rapamycin (mTOR), and AMP-activated protein kinase (AMPK) pathways after UMP supplementation (Fig. 6**C-D**). Protein expression profiling exhibited distinct species-specific signaling alterations. UMP-treated piglets showed higher protein abundances of PPARγ, and lower protein levels of AMPKα, p-AMPKα, p-mTOR, and phosphorylated eukaryotic translation initiation factor 4E-binding protein 1 (p-4EBP1) (*P* < 0.05; Fig. 8**A-B**). In addition, UMP-treated rats showed lower protein abundances of p-mTOR and p-4EBP1, and higher protein levels of signal transducer and activator of transcription 3 (STAT3), interleukin-6 (IL-6), interleukin-6 receptor (IL-6R), soluble interleukin-6 receptor (sIL-6R), and glycoprotein 130 (GP130) (*P* < 0.05; Fig. 8**C-D**).

## 4 Discussion

Piglets are widely recognized as an excellent model for studying early-life nutrition due to their physiological, metabolic, and developmental similarities to human infants, particularly in nutrient metabolism (Charton et al., 2024; He et al., 2025). The high-lactose rat model serves as a complementary tool to evaluate metabolic adaptation under nutritional challenge (Teichenné et al., 2023). This dual-model design enables a comprehensive assessment of hepatic metabolic alterations linked to UMP supplementation under high-lactose conditions.

### 4.1 Pyrimidine nucleotides: Milk supply and hepatic synthesis

Milk nucleotide composition varies across species and lactation stages. In both porcine and human milk, pyrimidine nucleotides (e.g., UMP) are abundant in colostrum and dynamically alter throughout lactation (Mateo et al., 2004; Mateos-Vivas et al., 2015). Consistent with these reports, abundant pyrimidines including UMP and CMP were predominant in early sow colostrum and declined thereafter, whereas purine nucleotides showed opposite trends. Serum uracil levels in suckling piglets also decreased in an age-dependent manner during lactation.

Hepatic pyrimidine homeostasis is maintained by de novo synthesis and salvage pathways (Fustin et al., 2012). The progressive downregulation of hepatic pyrimidine metabolism-related genes across lactation coincided with both postnatal physiological maturation of piglets and dynamic shifts in milk nutrient composition. Given the observational nature of this study, we cannot fully separate intrinsic developmental effects from the potential influences of milk lactose and UMP. Accordingly, these parallel changes are described as covariation patterns rather than nutrient-driven regulatory responses. Reduced milk nucleotide concentrations in late lactation may partially relate to elevated mammary tissue utilization (Kim et al., 1999), and these temporal trends reflect associations between milk nutrient levels and offspring hepatic metabolism. Differences in sampling time, genetics and feeding conditions lead to inconsistent milk nucleotide results across studies (Hodgkinson et al., 2022; Yu et al., 2025), so relevant cross-study extrapolation should be made cautiously.

In this time-series observational lactation model, alterations in milk components, serum metabolites and hepatic profiles coincided with piglet physiological maturation and lactation progression. Owing to the study design limitations, developmental effects cannot be disentangled from fluctuations in milk lactose and endogenous UMP. All observed metabolic trends represent synchronous covariation rather than causal regulation, reflecting combined physiological and dietary influences instead of isolated UMP- or lactose-specific metabolic responses.

### 4.2 Effect of UMP on glucose metabolism and hepatic glycogen storage under high-lactose conditions

Consistent with previous study (Theil et al., 2014), mature milk contains higher lactose levels than colostrum. Lactation progression was accompanied by broad metabolic adaptation in suckling piglets, marked by modulated systemic glucose and lipid utilization. Higher fructose 1-phosphate, citric acid and isocitrate, together with reduced galactose and galactaric acid, were associated with altered TCA cycle activity and changes in galactose, fructose and mannose metabolism. Hepatic glycogen gradually declined over lactation, while intrahepatic glucose increased after postnatal day 7, showing temporal covariation in hepatic glucose and glycogen levels during lactation progression. Although TCA-related genes and *G6PC* were transcriptionally suppressed, key gluconeogenic genes were upregulated at postnatal day 14. This divergent transcriptional pattern reflects distinct temporal alterations in glucose oxidation and gluconeogenic gene expression during neonatal development. The Leloir pathway also displayed clear developmental regulation (Holden et al., 2003). Persistent downregulation of *GALK* and *GALE* alongside transient *GALT* upregulation was accompanied by reduced circulating galactose metabolites, showing synchronous phenotypic and transcriptomic variation. Thus, the shifting milk nutrient profile was associated with systemic metabolic adaptation in suckling piglets, including changes in TCA cycle activity, hepatic glycogen mobilization, a transcriptional shift toward gluconeogenesis, and a progressive decline in galactose metabolism during lactation.

In tandem with these metabolic alterations, lactation progression coincides with coordinated reductions in milk pyrimidine nucleotides and hepatic de novo pyrimidine synthesis in suckling piglets. As a central pyrimidine intermediate, supplemental UMP was associated with distinct carbohydrate metabolism across both piglet and rat models. In weaned piglets, UMP was correlated with higher serum sucrose, glucose, and succinic acid levels. In rats, UMP was associated with hepatic TCA cycle intermediates (succinic acid) and glucuronate pathway metabolites (D-glucuronic acid). Accumulated D-glucuronic acid may reflect increased UDP-glucose production for glycogen synthesis (Yang et al., 2024), while elevated succinic acid may indicate higher TCA cycle flux. These observations are consistent with recent evidence linking pyrimidine metabolism to glucose homeostasis (Choi et al., 2025), and further support the correlation between nucleotide abundance and carbohydrate metabolism.

Hepatic glucose homeostasis depends on the coordination of gluconeogenesis, the TCA cycle, and the pentose phosphate pathway (Gao et al., 2026; Kajani et al., 2024). In agreement with metabolomic data, transcriptomic analysis identified significant enrichment of glycolysis/gluconeogenesis, galactose metabolism and pentose phosphate pathways associated with UMP. Specifically, UMP was associated with higher expression of core genes governing glucose uptake and galactose metabolism, alongside changes in activities of gluconeogenic and Leloir pathway enzymes across both models. These molecular and biochemical changes may reflect with higher gluconeogenic potential and higher conversion of galactose to UDP-glucose. Collectively, these observations suggest that UMP is associated with altered gluconeogenic activity and changes in galactose metabolic flux, which may contribute to the maintenance of hepatic glucose homeostasis and glycogen accumulation.

### 4.3 Effect of UMP on lipid metabolism and hepatic TG storage under high-lactose conditions

Mature milk contained higher fat levels than colostrum, consistent with previous findings (Theil et al., 2014). Increased lipid supply from mature milk coincides with higher systemic lipid turnover in neonatal piglets, supporting energy expenditure and rapid postnatal tissue growth. In the present study, UMP supplementation was correlated with consistent hepatic TG accumulation across both porcine and rodent models under high-lactose conditions, suggesting a conserved cross-species metabolic adaptation to UMP intervention and a possible correlative association between pyrimidine nucleotide availability and adaptive hepatic TG metabolism under modified nutritional status (Gao et al., 2021; Gao et al., 2022; Liu et al., 2021). Notably, such UMP-associated lipid accumulation occurred without hepatic ultrastructural impairment or aberrant liver function biomarkers, suggesting a non-pathological energy storage adaptation rather than pathological lipid over-deposition. Transcriptomic profiling further identified changes in hepatic lipid partitioning at the molecular level: UMP intervention was associated with higher expression of lipogenic genes and lower expression of genes related to lipolysis and fatty acid transport. Despite consistent TG accumulation, UMP was associated with species-specific fatty acid remodelling across models, with increased hepatic SFAs and MUFAs in piglets and elevated long-chain PUFAs in rats. These divergent lipid profiles are likely attributable to inherent interspecific differences in the expression and activity of fatty acid desaturases and elongases, consistent with previously documented disparities in hepatic n-3 and n-6 PUFA deposition between porcine and rodent models (Komprda et al., 2017). These species-specific differences are presented as phenotypic observations without forcing a unified mechanistic framework.

### 4.4 Effect of UMP on hepatic signaling pathways under high-lactose conditions

Hepatic carbohydrate and lipid metabolism are regulated by a complex network of interconnected signaling pathways (Del Piano et al., 2024). Consistent with metabolic phenotypic changes, transcriptomic profiling suggested that UMP supplementation was associated with changes of PPAR signaling, mTOR, AMPK, and IL-6-mediated signaling. UMP intervention was correlated with lower hepatic AMPK and p-AMPK abundance and altered mTOR/4EBP1 axis profiles. Given that the mTOR/4EBP1 axis is a well-characterized regulator of de novo pyrimidine synthesis (Ben-Sahra et al., 2013; Robitaille et al., 2013), this study suggests a potential feedback association between UMP abundance and mTOR-mediated endogenous nucleotide synthesis. Moreover, UMP supplementation was associated with higher of the IL-6, IL-6R, sIL-6R, and GP130 protein levels, which may integrate with AMPK signaling to coordinate hepatic glycogen metabolism and lipid utilization (Bertholdt et al., 2018; Kistner et al., 2022; MacDonald et al., 2003). Collectively, these observations suggest that changes in the AMPK/mTOR axis and IL-6 signaling are associated with UMP-related changes of lactose-associated hepatic carbohydrate and lipid energy metabolism. These findings are correlative and do not establish causality; functional validation is required to determine whether these signaling changes mediate the observed metabolic effects.

### 4.5 Limitations

This study has several limitations. First, milk contains a complex mixture of nutrients, and non-lactose components may also influence neonatal metabolic phenotypes. Therefore, the observed correlation between milk lactose and offspring hepatic metabolism cannot be interpreted as direct causation, and further controlled feeding trials are required to clarify the specific effects of lactose. Second, although two complementary animal models were used to enhance result robustness, inherent interspecies differences in lactose tolerance, hepatic lipid metabolism and nucleotide homeostasis between pigs and rats may limit the cross-species generalizability of our findings. Third, experimental piglets were matched to litter-average body weight at grouping. This strategy aimed to reduce individual physiological variation and improve statistical sensitivity, whereas it may cause selection bias and compress natural biological variation, thus limiting the extrapolation of current findings to piglets with varied body weight status. Fourth, the sample sizes for transcriptomic and ultrastructural analyses were relatively small. Though consistent with common exploratory research standards, they may reduce statistical reliability. Larger sample cohorts are needed to validate these results in future studies. Fifth, the 35% high-lactose diet in rats is a model of lactose intolerance and is supraphysiological. This artificial challenge was used to assess UMP-associated responses under nutritional stress; thus, the findings are proof-of-principle and not directly translatable. Finally, all transcriptomic and metabolomic data presented in this study are correlative. In the absence of targeted functional validation, the pathway enrichment and molecular mechanistic interpretations remain exploratory and cannot confirm direct regulatory interactions.

## 5 Conclusions

This study provided a comprehensive characterization of metabolic adaptations in suckling piglets during postnatal development and showed that UMP supplementation was associated with higher hepatic energy storage in both weaned piglets and rats. UMP supplementation was associated with higher hepatic glycogen and triglyceride deposition, together with changes in gluconeogenic- and lipogenic-related enzyme activities and gene expression. These metabolic changes coincided with alterations in AMPK/mTOR, PPARγ, and IL-6 signaling components.

## CRediT authorship contribution statement

**Lu-min Gao:** Writing – original draft, Methodology, Investigation, Data curation, Conceptualization. **Lu Liu:** Software, Investigation, Data curation. **Xu-dong Yang:** Investigation, Data curation. **Ci-min Long:** Methodology, Investigation. **Xin Wu:** Writing – review & editing, Methodology, Funding acquisition, Conceptualization.

## Declaration of competing interest

The authors declare that they have no known competing financial interests or personal relationships that could have appeared to influence the work reported in this paper.

## Data Availability

The untargeted metabolomics data for weaned and suckling piglets are available in the MassIVE repository under accession numbers MSV000101466 (weaned piglets; https://doi.org/10.25345/C5028PT1R) and MSV000101467 (suckling piglets; https://doi.org/10.25345/C5V980571). The raw RNA sequencing data generated in this study have been deposited in the NCBI BioProject under accession number PRJNA1450851 (project link: https://submit.ncbi.nlm.nih.gov/subs/sra/SUB16099411/overview).
