# Supplementary material for: Enhanced lactose utilization from milk enriched in Uridine-5’-monophosphate drives hepatic energy storage in young mammals
Source: Food Chem (Oxf). 2026 Jun 1;13:100427. doi: 10.1016/j.fochms.2026.100427 (PMC13266238; doi:10.1016/j.fochms.2026.100427)
Supplement: Supplementary file 1 — Supplementary material [file mmc1.docx]

**Table S1. Ingredients and composition of the basal diet for sows (as-fed basis)**

| Ingredients | Content (%) | Nutrient levels^2^ | Content |
| --- | --- | --- | --- |
| Corn | 64.35 | ME, MJ/kg | 13.40 |
| Soybean meal | 24.30 | DM | 86.77 |
| Steam fish meal | 3.00 | CP | 15.00 |
| Soybean oil | 2.50 | CF | 4.50 |
| Glucose | 1.00 | Ash | 5.50 |
| CaHPO4 (16.5%) | 1.53 | Ca | 0.85 |
| Calcium bicarbonate | 1.09 | Total P | 0.66 |
| NaCI | 0.50 |  |  |
| _L_-Threonine (98.5%) | 0.07 |  |  |
| _DL_-Methionine (98.5%) | 0.08 |  |  |
| _L_-lysine HCI (70%) | 0.38 |  |  |
| Vitamin-mineral premix^1^ | 1.20 |  |  |
| Total | 100.00 |  |  |

^1^ The vitamin-mineral premix provided the following per kilogram of diets:antioxidant 100 mg, sweetening agent 200 mg, 6,000 IU of vitamin A, 3,000 IU ofvitamin D3, 20 IU of vitamin E, 1.8 mg of vitamin K3, 2.0 mg of thiamine, 6.0 mg ofriboflavin, 4.0 mg of pyridoxine, 0.02 mg of vitamin B12, 26.0 mg of niacin, 18.0 mg ofpantothenic acid, 3.2 mg of folic acid, 0.4 mg of biotin, 20 mg of Cu as CuSO4 5H2O,100 mg of Zn as ZnSO4 H2O, 50 mg of Mn as MnSO4 H2O, 1.2 mg of I as KI, 0.30 mg ofSe as Na2SeO3. Feed carrier was zeolite powder.

^2^ Nutrient levels were calculated values.

**Table S2. Ingredients and nutrient levels of basal milk of weaned piglet (%, air-dry basis)**

| Ingredients | Content (%) | Nutrient levels ^2^ | Content |
| --- | --- | --- | --- |
| Skimmed milk powder | 85.00 | DE (MJ kg-1) | 14.65 |
| Dried whey | 5.00 | CP | 20.50 |
| Glucose | 2.50 | Ca | 8.4 mg/L |
| Plasma proteins | 3.50 | Total P | 5.59 mg/L |
| Premix ^1^ | 4.00 | SID Lys | 1.45 |
| Total | 100.00 | SID Met | 0.48 |
|  |  | SID Trp | 0.30 |
|  |  | SID Try | 0.29 |

^1^ The premix provided the following per kg of diets: vitamin A1, 500 IU; vitamin D3, 200 IU; vitamin E, 85 IU; D-pantothenic acid, 35 mg; vitamin B2, 12 mg; folic acid, 1.5 mg; nicotinic acid, 35 mg; vitamin B, 13.5 mg; vitamin B6, 2.5 mg; biotin, 0.2 mg; vitamin B12, 0.05 mg; copper (as copper sulfate), 15 mg; ferrum (as ferrous sulfate), 100 mg; manganese (as manganese sulfate), 20 mg; iodate (as calcium iodate), 1.0 mg; selenium (as sodium selenite), 0.35 mg; cobalt (as cobalt sulfate), 0.2 mg; and chromium (as chromium picolinate), 0.2 mg.

^2^ Ca and TP are measured values, and the rest are calculated values.

**Table S3. Composition of experimental diets for Sprague-Dawley rat**

| Ingredient | Content (%) | kcal/100g ^3^ |
| --- | --- | --- |
| Basal diet (AIN-93G) |  |  |
| Casein | 20.00 | 80.00 |
| Corn starch | 4.75 | 19.00 |
| Lactose | 35.00 | 140.00 |
| Sucrose | 10.00 | 40.00 |
| Dextrinized cornstarch | 13.20 | 52.80 |
| Soybean oil | 7.00 | 63.00 |
| Alphacel (non-nutritive bulk) | 5.00 | - |
| AIN-93G mineral mix ^1^ | 3.50 | - |
| AIN-93-VX vitamin mix ^2^ | 1.00 | - |
| L-Cystine | 0.30 | - |
| Choline bitartrate | 0.25 | - |
| Total | 100.00 |  |

^1^ The AIN-93G mineral mix containing calcium carbonate (357 g/kg), monopotassium phosphate (250 g/kg), potassium citrate monohydrate (28 g/kg), sodium chloride (74 g/kg), potassium sulfate (46.6 g/kg), magnesium oxide (24 g/kg), ferric citrate (6.06 g/kg), zinc carbonate (1.65 g/kg), manganese carbonate (0.63 g/kg), copper carbonate (0.30 g/kg), potassium iodate (0.01 g/kg), sodium selenate (0.01 g/kg), ammonium molybdate tetrahydrate (0.008 g/kg), sodium metasilicate nonahydrate (1.45 g/kg), chromium potassium sulfate dodecahydrate (0.275 g/kg), lithium chloride (0.017 g/kg), boric acid (0.082 g/kg), sodium fluoride (0.064 g/kg), nickel carbonate (0.032 g/kg), ammonium vanadate (0.007 g/kg), and powdered sucrose (209.806 g/kg).

^2^ The AIN-93-VX vitamin mix containing nicotinic acid (3.00 g/kg), D-calcium pantothenate (1.60 g/kg), pyridoxine HCl (0.70 g/kg), thiamine HCl (0.60 g/kg), riboflavin (0.60 g/kg), folic acid (0.20 g/kg), D-biotin (0.02 g/kg), vitamin B12 (0.1% in mannitol, 2.50 g/kg), α-tocopherol powder (250 U/g, 30.00 g/kg), vitamin A palmitate (250,000 U/g, 1.60 g/kg), vitamin D3 (400,000 U/g, 0.25 g/kg), phylloquinone (0.075 g/kg), and powdered sucrose (959.655 g/kg).

^3^ Nutrient levels were calculated values.

**Table S4. Primer sequences**

| **Item** | | **Accession No.** | **Nucleotide sequence of primers (5′-3′)** | **Size, bp** |
| --- | --- | --- | --- | --- |
| *PCK1* | | NM_001123158.1 | F:TCAGCACGACTCCAGCCTTCA | 122 |
|  |  |  | R:GCTCAAGCAGTCTGGGCATTCT |  |
| *FBP1* | | NM_213979.1 | F:TCCACCGCACGCTGGTCTAT | 129 |
|  |  |  | R:CCAGTCCTCCTGCCTTCTCCAT |  |
|  |  |  | R:GGAAGCCGATGGTGTTGGAGAA |  |
| *CS* | | XM_021091147.1 | F:TCTCAGCTCAGTGCAGCCATTACA | 145 |
|  |  |  | R:TCTCAGCTCAGTGCAGCCATTACA |  |
| *ICDH β* | | [XM_021077332.1](https://www.ncbi.nlm.nih.gov/nuccore/XM_021077332.1) | F:TGTGGTTCCTGGTGAGAG | 149 |
|  |  |  | R:CGAGATTGAGATGCCGTAG |  |
| *G6PC* | | NM_001113445.1 | F:AAGCCAAGCGAAGGTGTGAGC | 165 |
|  |  |  | R:GGAACGGGAACCACTTGCTGAG |  |
|  |  |  | R:CTTCTGGGGCTTCTTGAATGTC |  |
| *GLUT1* | | XM_021096908.1 | F:GCAGGAGATGAAGGAGGAGAGC | 258 |
|  |  |  | R:ACGAACAGCGACACGACAGT |  |
| *GALE* | | XM_021095529.1 | F:AGTGCTGCTGCGATACTTCAACC | 143 |
|  |  |  | R:TGCCAAAGACATTCAGTGCCTCTC |  |
| *GALT* | | XM_005668044.3 | F:CATGGTTGGCTACGAAATGCTTGC | 136 |
|  |  |  | R:GCTGCTGTCTCCTTGTCCTTCTG |  |
| *GALK1* | | XM_003131193.2 | F:GGAGAGCCTTCCTGGAGGAGTTC | 146 |
|  |  |  | R:GCCCACGAGCACAGTCACAAG |  |
|  |  |  | R:TAGGTGCACTTGATGCAGGG |  |
| *OGT* | | NM_001039748.2 | F:TTGACTGCCCCTCTATGCTG | 131 |
|  |  |  | R:CCTGGGATTTCATTTCTGCTG |  |
| *GSK3B* | | XM_021068187.1 | F:AGGTCCTAGGGACACCAACA | 161 |
|  |  |  | R:TGTGTACTCCAACAGACGGC |  |
| *FABPPm* | | NM_213928.1 | F:ATGGGCTTATACGGTGAGCG | 127 |
|  |  |  | R:CGTTGACAGGAGGGTTGGAA |  |
|  |  |  | R:AACCACTGTCTTGACCTTCTCC |  |
| *FAT/CD36* | | XM_021102279.1 | F:CTGGTGCTGTCATTGGAGCAGT | 161 |
|  |  |  | R:CTGTCTGTAAACTTCCGTGCCTGTT |  |
| *FATP1* | | NM_001083931.1 | F:GGTGCTTGTGGCTTCAACAG | 276 |
|  |  |  | R:CACCAGCACGTCACCTGATA |  |
|  |  |  | R:GACCGGCTCTCCATAGACAA |  |
| *FAS* | | NM_001099930.1 | F:GTCCTGCTGAAGCCTAACTC | 206 |
|  |  |  | R:TCCTTGGAACCGTCTGTG |  |
| *ACC* | | XM_021066238.1 | F:GGAGACAAACAGGGACCATTACA | 144 |
|  |  |  | R:CAGGGACTGCCGAAACATC |  |
| *FADS1* | | NM_001113041.1 | F: GTCACTGCCTGGCTCATTCT | 155 |
|  |  |  | R:AGGTGGTTCCACGTAGAGGT |  |
| *FADS2* | | NM_001171750.1 | ACGGCCTTCATCCTTGCTAC | 144 |
|  |  |  | GTTGGCAGAGGCACCCTTTA |  |
| *ELOVL-5* | | XM_021098832.1 | F:TACCACCATGCCACTATGCT | 102 |
|  |  |  | R: GACGTGGATGAAGCTGTTGA |  |
| *ELOVL-2* | | XM_013977421.1 | F:ATTCTTCACCACCAGCGAGG | 131 |
|  |  |  | R:TGCCTGGCTGTTATCACTCG |  |
| *SCD* | | XM_021072070.1 | F:TTGCTCTGGGCGTTTGC | 92 |
|  |  |  | R:CGAGCTTTGTAAGTTCGGTGACT |  |
| *ATGL* | | XM_021076533.1 | F:ATGGTGCCCTACACGCTG | 177 |
|  |  |  | R:GCCTGTCTGCTCCTTTATCC |  |
| *CPT-1α* | | NM_001129805.1 | F:AGCGAGTGTGCCAGATACAAA | 118 |
|  |  |  | R:AGCGAGTGTGCCAGATACAAA |  |
| *CAD* | | XM_021087696.1 | F:GATGGGAACCCTTGATGGCA | 129 |
|  |  |  | R:TGGCCCACTAAAGAGTGCAG |  |
| *CTPS2* | | XM_021080709.1 | F:ACTCCAGGCGATTTCTTGGG | 154 |
|  |  |  | R:CACAGGAACACGGGCATTTG |  |
| *CMPK1* | | XM_003128011.6 | F:CACGTCCTAGGCCTTAGCTTC | 93 |
|  |  |  | R:GAGGACGAACACGACCTTCG |  |
| *CTPS1* | | XM_003128105.4 | F:CACCAATCAGGGGTCAGCAG | 134 |
|  |  |  | R:TGGAGGTCACGTGTAAACCG |  |
| *UMPS* | | XM_021071253.1 | F:ATTTGGGAGCTTCGTGCTGA | 134 |
|  |  |  | R:TGATCCCTGCATTTTGGGCA |  |
| *UCK1* | | XM_003122250.4 | F:CTCTTCGTGGACACCGACTC | 113 |
|  |  |  | R:GGCTTGACGAAGGTGGTGTA |  |
| *UPP2* | | XM_021076463.1 | F:TGGACGATACCATCACCCGA | 155 |
|  |  |  | R:TGCTCCATCTAGTCGACCCT |  |
| *CDA* |  | NM_001244385.1 | F:ATTGCCATCGCCAGTGATCT  R:CCATCCGGCTTGGTCATGTA | 113 |
| *β-actin* | | *XM_003357928.4* | F: CGTTGGCTGGTTGAGAATC | 132 |
|  |  |  | R: CGGCAAGACAGAAATGACAA |  |

**Table S5. Primary antibodies used for immunoblotting**

| **Target** | **Catalog Number** | **Source** | **Dilution** |
| --- | --- | --- | --- |
| GAPDH | 2118 | Cell Signaling Technology | 1:1000 |
| PPARα | ab233078 | Abcam | 1:2000 |
| PPARγ | ab209350 | Abcam | 1:1000 |
| AMPKα | 2535S | Cell Signaling Technology | 1:1000 |
| p-AMPKα | 5831S | Cell Signaling Technology | 1:1000 |
| p-mTOR | 5536S | Cell Signaling Technology | 1:1000 |
| mTOR | 2974S | Cell Signaling Technology | 1:1000 |
| Raptor | 2280S | Cell Signaling Technology | 1:1000 |
| p-4EBP1 | SC-293124 | Santa Cruz Biotechnology | 1:500 |
| 4-EBP1 | SC-9977 | Santa Cruz Biotechnology | 1:500 |
| p-P70S6Kα | SC-377529 | Santa Cruz Biotechnology | 1:500 |
| IL6 | 12153 | Cell Signaling Technology | 1:1000 |
| IL6R | A1570 | ABclonal | 1:1000 |
| GP130 | A18036 | ABclonal | 1:1000 |
| STAT3 | 12640 | Cell Signaling Technology | 1:1000 |
| p-STAT3 | 9145 | Cell Signaling Technology | 1:1000 |

**Table S6. List of abbreviations used in this study**

| **Abbreviation** | **Full name** |
| --- | --- |
| UMP | Uridine-5'-monophosphate |
| CMP | Cytidine Monophosphate |
| GMP | Guanosine Monophosphate |
| IMP | Inosine Monophosphate |
| AMP | Adenosine Monophosphate |
| GLU | Glucose |
| TC | Total cholesterol |
| TG | Triglyceride |
| FAMEs | Fatty acid methyl esters |
| CAD | Carbamoyl-phosphate synthetase 2, aspartate transcarbamylase, and dihydroorotase |
| UMPS | Uridine monophosphate synthetase |
| CDA | Cytidine deaminase |
| UPP2 | Uridine phosphorylase 2 |
| UCK1 | Uridine-cytidine kinase 1 |
| UCP2 | Uncoupling protein 2 |
| CTPS1 | CTP synthase 1 |
| CTPS2 | CTP synthase 2 |
| CMPK1 | CMP kinase 1 |
| FBP | Fructose-1, 6-bisphosphate |
| PK | Pyruvate kinase |
| HK | Hexokinase |
| PEPCK | Phosphoenolpyruvate carboxykinase |
| PC | Pyruvate carboxylase |
| ICDHm | Isocitrate dehydrogenase |
| GALK | Galactokinase |
| GALT | Galactose-1-phosphate uridylyltransferase |
| GDH | Glucose dehydrogenase, |
| UDP-GD | UDP-glucose dehydrogenase |
| GALE | UDP-galactose 4-epimerase, |
| UGP | UDP-glucose pyrophosphorylase. |
| GLUT1 | Glucose transporter protein 1. |
| ACC | Acetyl-CoA carboxylase |
| FAS | Fatty acid synthase, |
| FADS1 | Fatty acid desaturase 1, |
| FADS2 | Fatty acid desaturase 2, |
| ELOVL5 | Fatty acid elongase 5 |
| ELOVL2 | Fatty acid elongase 2 |
| SCD | Stearoyl-CoA desaturase |
| ATGL | Adipose triglyceride lipase |
| CPT-1a | Carnitine palmitoyltransferase 1 a |
| FABPpm | Fatty acid-binding protein |
| FAT/CD36 | Fatty acid transporter/CD36 |
| FATP1 | Fatty acid transport protein 1 |


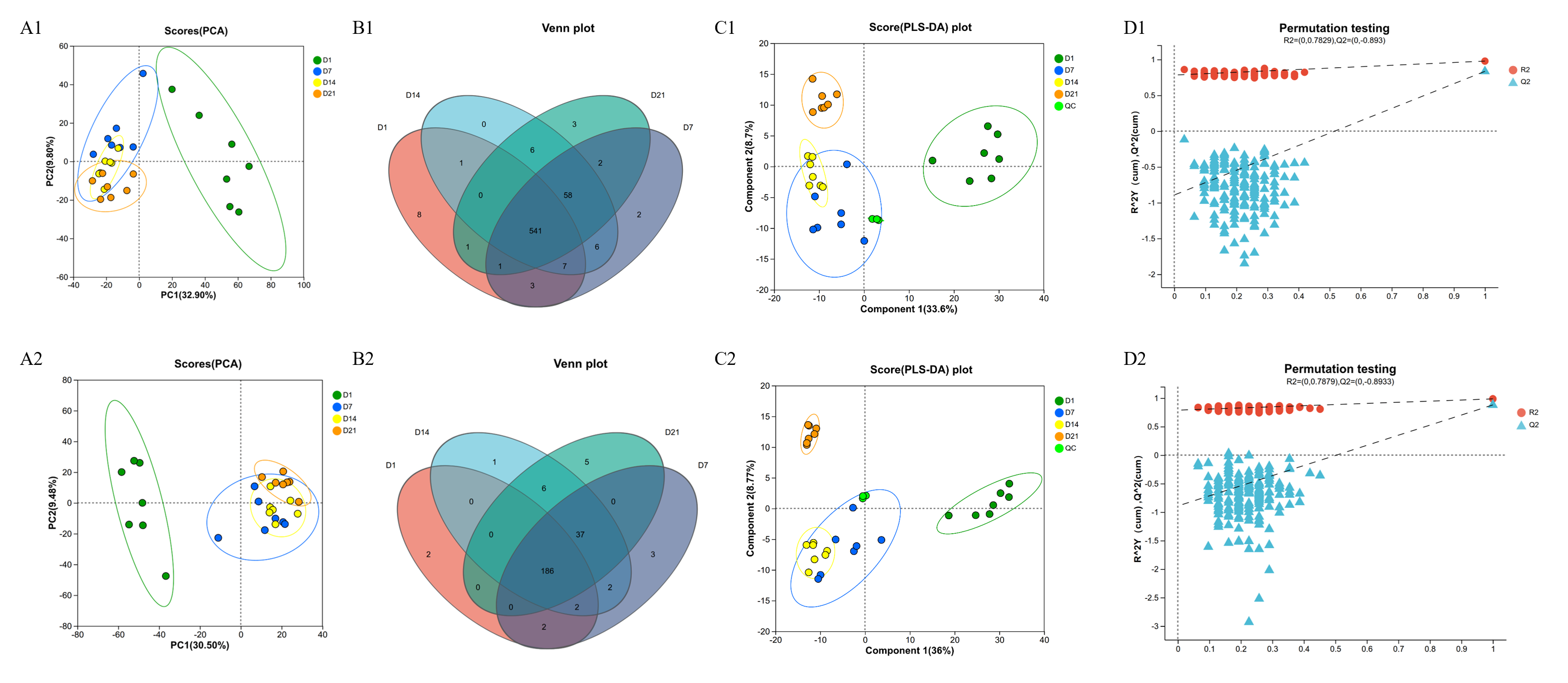


**Fig S1. Multivariate analysis of metabolomics data in serum of suckling piglets.** (A) PCA scores plot (positive ion mode, A1; negative ion mode, A2). (B) Volcano plot (positive mode, B1; negative mode, B2). (C) OPLS-DA scores plot (positive mode, C1; negative mode, C2). (D) Permutation test of the OPLS-DA model (200 tests) (positive mode, D1; negative mode, D2). Model quality parameters (R²X, R²Y, Q²) are indicated.


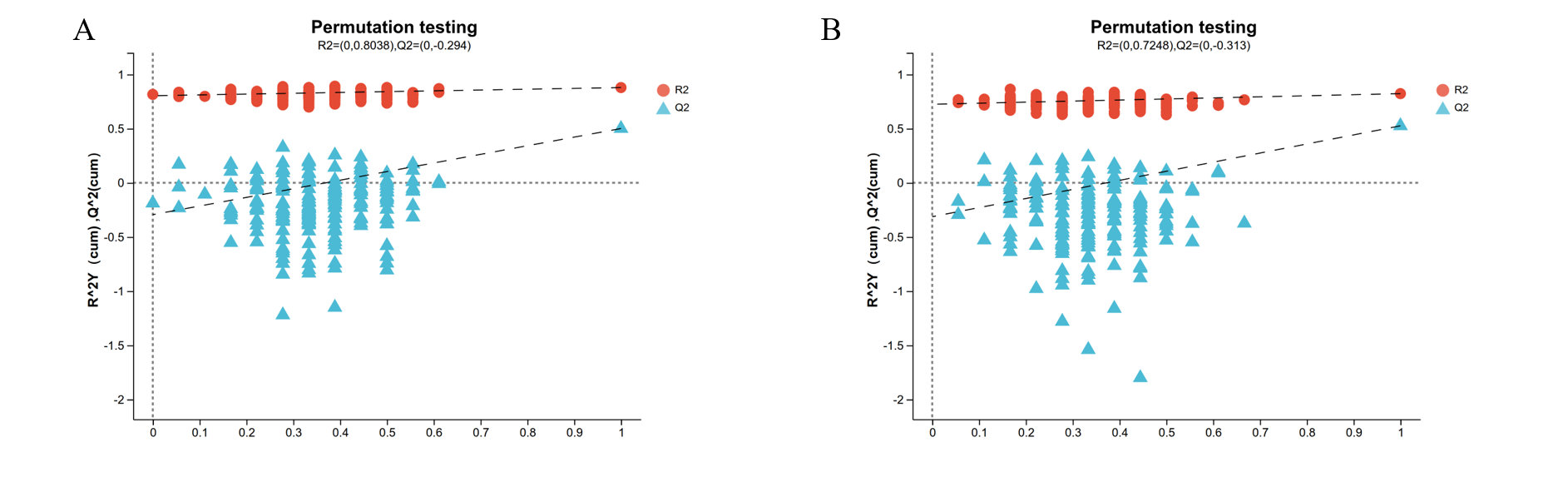


**Fig S2. OPLS-DA model validation for serum metabolomics in weaned piglets.** Permutation test results (200 tests) are shown for positive (A) and negative (B) ion modes, with R²X, R²Y, and Q² values indicated.

**Fig S3. Serum ALT and AST levels in weaned piglets.** Serum ALT and AST activities of weaned piglet were measured using an automatic biochemical analyzer (Synchron CX Pro, Beckman Coulter, USA) with commercial kits (Roche Diagnostics, China). Data are presented as mean ± SEM (n = 8). Statistical significance was determined by unpaired Student’s t-test, with **P* < 0.05 considered significant.
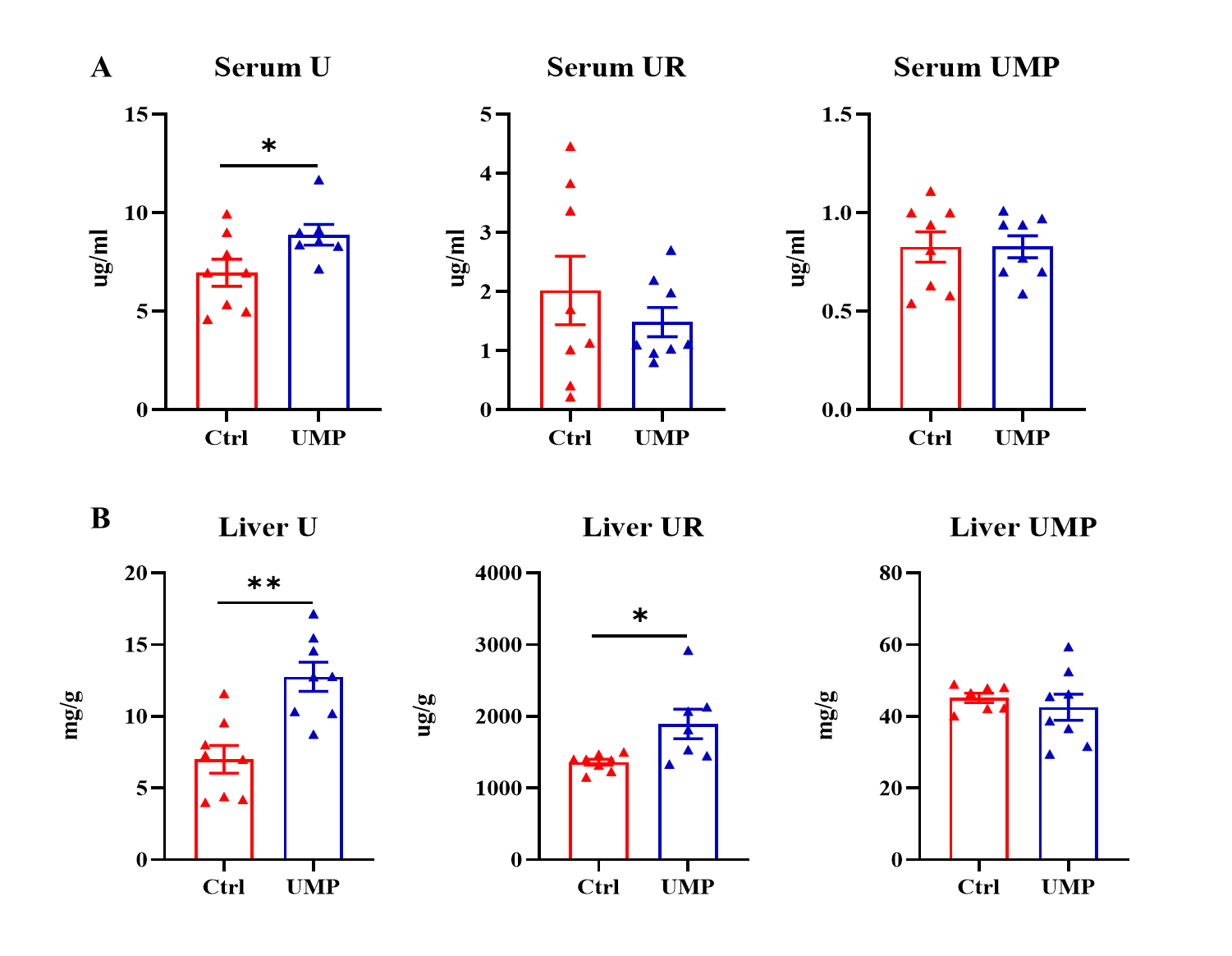
 **Fig S4. Effects of UMP on nucleotide metabolism in weaned piglets.** (A) Serum levels of uracil (U), uridine (UR), and uridine monophosphate (UMP). (B) Hepatic levels of uracil (U), uridine (UR), and uridine monophosphate (UMP). Pyrimidine nucleotides were quantified by liquid chromatography-tandem mass spectrometry (LC-MS/MS) using a Triple TOF 6600 system (SCIEX, Framingham, MA, USA). Data are shown as mean ± SEM (n = 8). Statistical significance was determined by unpaired Student's t-test (**P* < 0.05, ***P* < 0.01).


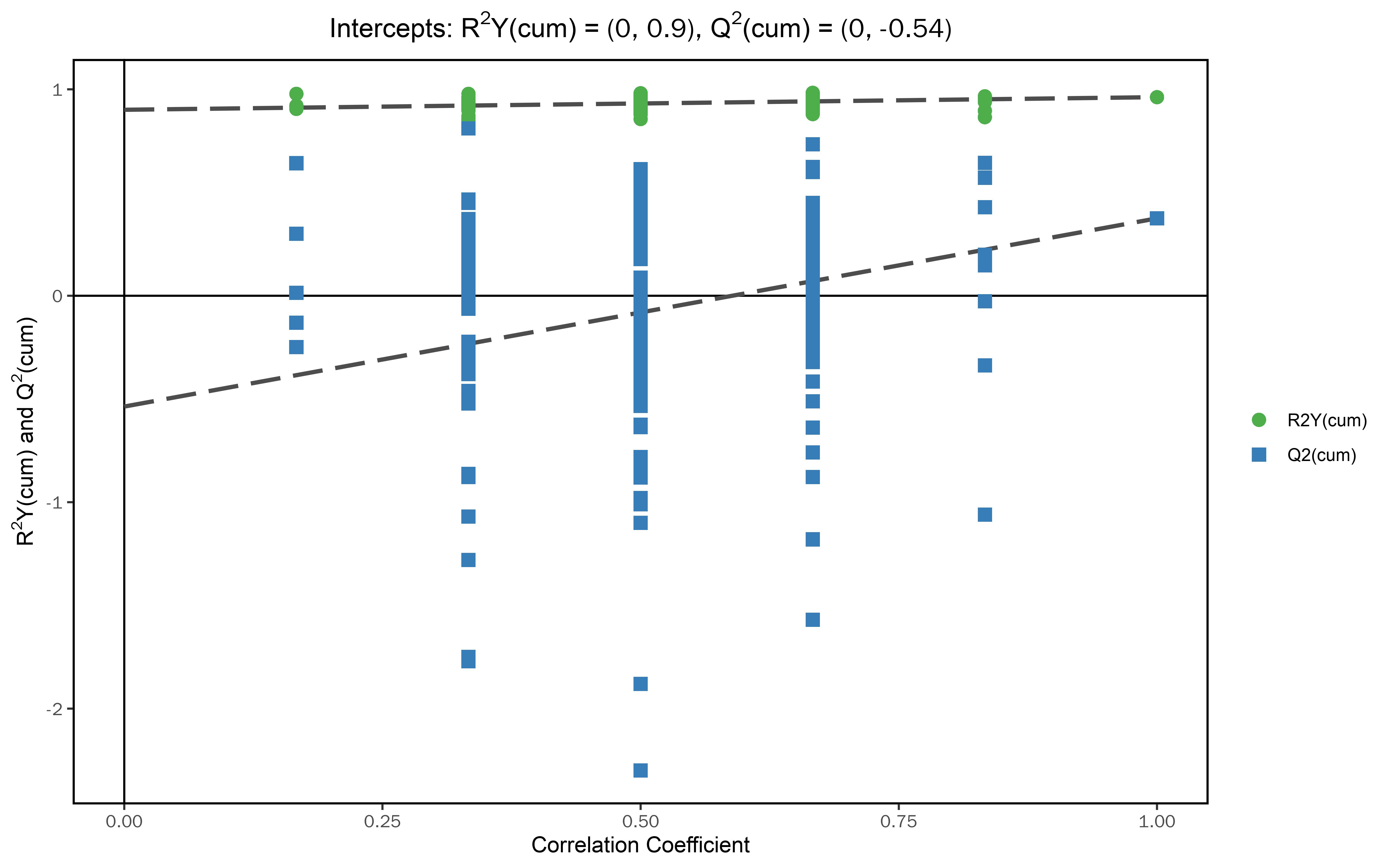


**Fig S5. OPLS-DA model validation for liver metabolomics in weaned rats.** Permutation test results (200 tests) are shown with R²X, R²Y, and Q² values indicated.
